# Genetic Mechanisms of Cold Signaling in Wheat (*Triticum aestivum* L.)

**DOI:** 10.3390/life12050700

**Published:** 2022-05-07

**Authors:** Qiangbo Liu, Xiang Zhang, Ying Hua Su, Xian Sheng Zhang

**Affiliations:** State Key Laboratory of Crop Biology, College of Life Sciences, Shandong Agricultural University, Tai’an 271018, China; liuqiangbo@sdau.edu.cn (Q.L.); 15106616076@163.com (X.Z.)

**Keywords:** cold stress, wheat, hormonal, reactive oxygen species, epigenetic regulation

## Abstract

Cold stress is a major environmental factor affecting the growth, development, and productivity of various crop species. With the current trajectory of global climate change, low temperatures are becoming more frequent and can significantly decrease crop yield. Wheat (*Triticum aestivum* L.) is the first domesticated crop and is the most popular cereal crop in the world. Because of a lack of systematic research on cold signaling pathways and gene regulatory networks, the underlying molecular mechanisms of cold signal transduction in wheat are poorly understood. This study reviews recent progress in wheat, including the *ICE*-*CBF*-*COR* signaling pathway under cold stress and the effects of cold stress on hormonal pathways, reactive oxygen species (ROS), and epigenetic processes and elements. This review also highlights possible strategies for improving cold tolerance in wheat.

## 1. Introduction

Higher plants are sessile organisms that suffer from various environmental stresses throughout their life cycle, such as cold, heat, drought, and salinity. Cold stress is vital for limiting plant geographical distribution and influencing plant growth and development, and ultimately determines yield and quality [1,2]. After a long interaction with their environment, plants have evolved complex and sophisticated mechanisms to adapt to cold stress. Cold signals are transduced from the plasma membrane to the nucleus, leading to a series of cold-induced cellular responses and the induction of cold-responsive genes. The main cold-responsive genes in plants are *C-REPEAT BINDING FACTORs* (*CBFs*), *INDUCER of CBF EXPRESSION* (*ICE*), and *COLD-REGULATED* (*COR*) genes [3,4]. It has been established that the *ICE*-*CBF*-*COR* signaling pathway is a universal pathway associated with cold tolerance in plants [2,5,6]. In this pathway, *CBFs/DEHYDRATION-RESPONSIVE ELEMENT BINDING FACTOR 1s* (*DREB1s*) is rapidly induced by cold conditions. Additionally, CBFs/DREB1s proteins can bind to the promoter regions of *COR* genes to activate their transcription in *Arabidopsis* [7,8].

Low-temperature conditions seriously affect the growth and development of wheat grown in temperate regions [9]. Exposure to low temperatures changes various biochemical processes and induces membrane damage in wheat [9,10]. The effects of cold stress on wheat growth, development, and yield are determined not only by the degree and duration of low-temperature conditions but also by the growth stage in which the cold stress events occur [11]. Cold stress can significantly reduce the viable leaf area and the soluble carbohydrate accumulation, ultimately negatively affecting the final yield [12,13]. During the reproductive phase, wheat is susceptible to cold stress. The grain number will be decreased by low temperatures if stress occurs before anthesis [14]. Moreover, pollen tube elongation and gametophyte tissue development will be disrupted under cold stress, particularly in pollen tapetal cells, which can lead to pollen sterility [15,16]. Cold stress can alter sink-source distribution by increasing the accumulation of soluble carbohydrates to regulate grain filling in wheat [9]. In addition, low-temperature events could also happen during vegetative stages in wheat and are detrimental to wheat growth and development since they cause leaves to wilt [17]. The leaf mass ratio and relative growth rate are significantly increased under low-temperature conditions. Furthermore, the flag leaf size and wheat biomass both decrease under cold stress [18,19]. Biomass allocation is essential for grain yield formation under cold stress, and the appropriate allocation is responsible for reproductive growth proportion and yield formation [20]. Diploid and tetraploid wheat have large leaf areas and produce no or low yield under cold stress. In contrast, hexaploid wheats have relatively low leaf areas and higher rates of grain yield among these species [13].

Cold stress has been categorized into two primary groups: chilling stress (0–15 °C) and freezing stress (<0 °C), which depends on how the plants are affected [1,21]. The cellular and molecular responses of plants to cold stress have been intensively studied. At present, plants have acquired highly sophisticated systems to cope with cold stress. For instance, plants activate a series of biochemical and physiological changes in their cells, such as altering the transcription of cold-responsive genes, regulating hormone levels and responses, producing ROS to stimulate the accumulation of compatible osmolytes and antioxidants, and remodeling genome-wide epigenetic modifications [8,22]. Significant progress has been made over the past few decades in understanding how signaling pathways control cold stress responses in plants. However, current knowledge of the cold signal transduction pathway in wheat is limited. In this review, we summarize the most recent studies assessing cold stress response in wheat and highlight possible strategies for improving cold tolerance in wheat.

## 2. ICE-CBF-COR Signaling Pathway in Cold Stress

Plants in temperate regions, such as winter wheat, have evolved adaptive responses known as cold acclimation, where plants acquire freezing tolerance after prior exposure to low non-freezing temperatures [8]. It is well known that the *ICE*-*CBF*-*COR* signaling pathway is essential for cold acclimation [3]. In addition to *Arabidopsis*, the *ICE*-*CBF*-*COR* cascade has been identified in rice [23] and wheat [6,24].

**Figure 1:**
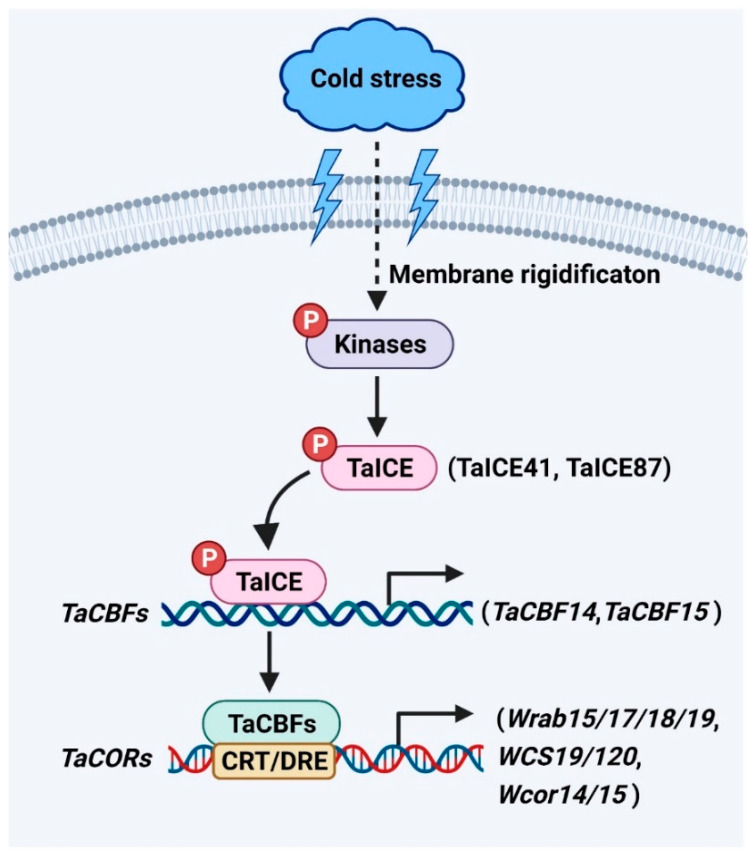
*ICE*-*CBF*-*COR* signaling pathway plays a vital role in wheat. Cold stress alters the fluidity of plasma membrane and activates protein kinases. Furthermore, kinases positively regulate cold tolerance in wheat by phosphorylating TaICE proteins, including TaICE41, TaICE87. TaICE directly binds to the promoters of *TaCBFs* to regulate its expression. Additionally, TaCBFs bind to the CRT/DRE sequence in the promoters of *TaCOR* genes, such as *Wrab15*, *Wrab17*, *Wrab18*, *Wrab19*, *WCS19*, *WCS120*, *Wcor14*, and *Wcor15*, for their transcription activation in response to cold stress.

*ICE* genes encode a class of MYC-like *bHLH* transcriptional factors upstream of the cold signaling pathway [25]. The C-terminal regions of *ICE* have highly conserved regions for specific interactions with downstream cold regulatory genes [5,24,25,26,27]. The homologs of ICE have been identified as TaICE41 and TaICE87 in wheat (Figure 1). Overexpression of *TaICE41* or *TaICE87* in *Arabidopsis* enhanced cold tolerance, suggesting the significance of ICE homologs in cold stress response [24]. HOS1 (HIGH EXPRESSION OF OSMOTICALLY RESPONSIVE GENE 1), an E3 ubiquitin ligase, reduces the stability of ICE1 protein by ubiquitination under cold stress [28]. In addition, the stability of ICE1 protein is enhanced by SUMO E3 ligase SIZ1 (SAP and Miz) through sumoylation in response to cold stress [29]. ICE1 is phosphorylated by the cold-activated protein kinase OPEN STOMATA 1 (OST1), resulting in weakened interaction between ICE1 and HOS1 to increase the stability of ICE1 under cold stress [30]. Furthermore, the stability of OsICE1 is up-regulated by OsMPK3 (MAP KINASE 3) through phosphorylation in rice in response to cold stress [31]. These results indicate that the posttranslational modification of ICE1 is crucial for its role in response to cold stress. However, whether TaICEs have similar regulatory mechanisms in wheat responses to cold stress needs further study.

*CBFs* (*CBF1*, *CBF2*, and *CBF3*), which belong to the AP2/ERF multi-gene family, can be activated by ICE in the cold signaling pathway of plants [3,25]. *CBFs* are key components for increasing the cold tolerance of plants [32,33,34]. The overexpression of *CBFs* in rice, maize, barley, wheat, and other plant species significantly enhances the cold tolerance of transgenic plants [35,36,37,38,39]. However, the *cbfs* triple mutant in *Arabidopsis* show reduced cold tolerance and larger biomass than wild type [40]. These results indicate *CBFs* may act to balance cold tolerance and plant growth. However, whether *CBFs* are important regulators of growth and cold tolerance to enhance the biomass of wheat requires further study. Several *CBF* genes have been characterized in *Triticeae* species, including 37 genes from hexaploid wheat [41], 20 genes from barley [36], 13 genes from *Triticum monococcum* [42], 11 genes from rye [43], ten genes from durum wheat [44], ten genes from *Aegilops biuncialis* [5], four genes from *Brachypodium distachyon* [45,46], and one gene from *Aegilops tauschii* [41]. TaCBF14 and TaCBF15, two wheat CBF transcription factors, play significant roles in cold stress response (Figure 1) [38]. Overexpression of *TaCBF14* or *TaCBF15* in barley enhances the expression of *HvCOR14b*, a cold-regulated gene in barley, increasing cold tolerance [38]. Additionally, *T. aestivum ABIOTIC STRESS-INDUCED DNA BINDING FACTOR a* (*TaAIDFa*) is markedly activated by cold stress [47]. Overexpression of *TaAIDFa* in *Arabidopsis* increases the transcription of the cold-regulated genes like *RD29A* and *COR15A* to enhance the cold tolerance of transgenic lines [47].

CORs generally refer to the protective substances encoded by cold-regulated genes. The protective substances such as osmolytes and cryoprotective proteins accumulate to facilitate cold acclimation and freezing tolerance [1,6]. CBFs are known to bind to the C-REPEAT/DEHYDRATION RESPONSIVE ELEMENT (CRT/DRE) sequence (TACCGCAT) in the promoters of *COR* genes for their transcription activation in response to cold stress [48,49]. The expression of ABA-dependent *COR* genes (*Wrab15/17/18/19*) and ABA-independent *COR* genes (*WCS19*, *WCS120*, *Wcor14*, and *Wcor15*) are significantly increased by cold stress in wheat (Figure 1) [50]. The expression of *DRE-BINDING PROTEIN 1* (*TaDREB1*), a wheat homolog of *Arabidopsis DREB2*, is elevated under cold stress [51]. The transcription of the *WHEAT COLD SPECIFIC 120* (*WCS120*) gene is activated by TaDREB1 and increases cold tolerance in winter wheat [52]. The expression of wheat *DREB2* (*WDREB2*), also a wheat homolog of *Arabidopsis DREB2*, is activated by cold [53]. The WDREB2 transcription factor directly affects the expression of wheat *COR* genes such as *Wrab19* in response to cold stress [53].

## 3. Cold Stress Influences Hormonal Responses

Plant hormones (Phytohormones), which function as small molecules to regulate various cellular processes and work as chemical messengers to communicate cellular activities, are produced in very low concentrations in higher plants [54]. Phytohormones are needed for plants to deal with abiotic stresses, including salinity, drought, and low temperature, by mediating a wide range of adaptive responses [55]. These phytohormones include auxin, abscisic acid (ABA), ethylene, cytokinins (CKs), gibberellins (GAs), jasmonic acid (JA), brassinosteroids (BRs), salicylic acid (SA), and strigolactones (SLs). In recent years, the phytohormone signaling pathway has been investigated by genetic and biochemical approaches, and a growing body of evidence indicates that the elements in hormonal signaling pathways contribute to regulating plant cold tolerance [33].

Auxin, a tryptophan derivative most commonly present in the form of indole-3-acetic acid (IAA), plays an essential role in plant development and cold stress response. The *YUCCA* genes encode the key rate-limiting enzymes in the auxin biosynthetic pathway and are involved in the regulation of plant growth and development. The transcript levels of *OsYUCCAs* are strongly induced by low temperatures; however, the expression of IAA catabolism-related genes, *Oryza sativa* *GRETCHEN HAGENs* (*OsGHs*), is down-regulated, resulting in significantly increased IAA content in rice under cold stress (Table 1) [56]. In colder/ambient temperatures, CLAVATA (CLV) peptide signaling promotes flower development by stimulating auxin-dependent growth. In contrast, at higher temperatures, *YUCCA* genes are activated to maintain flower development bypass CLV signaling [57,58,59]. There are 15 genes among 63 *TaYUCCAs* that are induced by drought and heat stress in wheat, though it is unclear whether the expression of these genes is regulated by cold stress. *Arabidopsis AUXIN RESPONSE FACTOR* (*ARF*) genes, which regulate the expression of auxin-responsive genes by binding to the auxin response element in their promoters, are up-regulated during cold acclimation (Table 1) [60]. In wheat, 46 genes from 69 *TaARFs* are also up-regulated in response to cold stress (Table 1) [61].

Abscisic acid (ABA) is the most important phytohormone due to its role in plant adaptation to biotic and abiotic stresses [62]. ABA-deficient mutants in *Arabidopsis* show defects in freezing tolerance, with the induced expression of *COR* genes, suggesting that ABA is involved in cold signaling [63,64]. Additionally, ABA contents are moderately decreased after cold treatment [30]. SUCROSR NON-FERMENTING 1-RELATED PROTEIN KINASE 2s (SnRK2s) are important protein kinases in ABA signaling, and their role in abiotic and biotic stress signaling has been extensively characterized in *Arabidopsis*. The SnRK2 homologs in wheat appear to play a critical role in cold signaling. PKABA1, the first SnRK2 protein identified in wheat, is rapidly induced in seedlings when ABA levels increase in response to cold stress [65]. Furthermore, the expression of *TaSnRK2.3*, *TaSnRK2.4*, and *TaSnRK2.8* can be induced by cold stress, suggesting that they are essential in cold signal transduction (Table 1) [66,67,68]. Overexpression of *TaSnRK2.3* or *TaSnRK2.8* in *Arabidopsis* increases cold tolerance, which is due to the increased expression of cold-responsive genes, and the enhanced accumulation of stress-associated metabolites such as proline [67]. Recent studies have identified 10 *SnRK2* homologs in wheat, and the expression of these genes is induced by cold stress [69]. Although ABA and cold signaling are closely related, it is unclear what the exact role of ABA in regulating plant cold stress responses is. Further work is needed to elucidate the molecular mechanisms of ABA when regulating cold signaling pathways.

Ethylene, a gaseous plant hormone, is important in various cellular and developmental processes, as well as during abiotic and biotic stress responses [70,71,72,73,74,75]. It is reported that cold stress can alter endogenous ethylene levels in many plant species. Cold stress inhibits ethylene production in *Arabidopsis* [76]; however, the ethylene levels are increased in winter rye under cold stress [77]. *T. aestivum ethylene-responsive factor 1* (*TaERF1*), the first member of the ERF gene family identified in wheat, is induced by cold stress (Table 1). Additionally, *TaERF1* overexpression can activate *COR* genes and improve freezing tolerance in transgenic *Arabidopsis* [78]. Pathogen-induced ethylene response factor 1 (TaPIE1) in wheat positively regulates freezing stresses by activating cold-regulated genes downstream of the ethylene signaling pathway and by modulating related physiological traits (Table 1) [79].

Gibberellins (GAs) play vital roles in abiotic stress response and adaptation. DELLA proteins are master regulators of GA-responsive growth and development [80]. Cold stress activates the expression of *GA 2-oxidase* genes to reduce the content of GA, resulting in the enhanced accumulation of DELLA proteins [81]. It is reported that overexpression of *CBFs* reduces the bioactive GA levels to suppress plant growth and flowering. *CBF1*-overexpression plants exhibit dwarfism and late-flowering phenotypes due to limited accumulation of bioactive GA [81]. Additionally, the *cbfs* mutants display impaired cold tolerance and larger architecture than the wild type after cold acclimation [40,82]. These results indicate that both the content and signal components of GA are related to cold signaling and CBFs may be associated with GA signaling to balance low-temperature adaption and growth. DELLAs act early in the cold signaling pathway as regulators of *GROWTH REGULATORY FACTORs* (*GRFs*). Cold-induced *CBF* genes are decreased in *GRF5*-overexpression lines, indicating that GRFs can repress *CBF* expression under cold stress (Table 1) [83]. Overexpression of *SLENDER RICE 1* (*SLR1*), a gene that encodes the rice DELLA protein, enhances chilling tolerance. When rice seedlings are subjected to chilling stress, the cold-induced *SLR1* (Table 1) releases the repressive effect of OsGRF6 on *OsGA2ox1*. The increased *OsGA2ox1* expression then decreases the active GA levels to enhance rice chilling tolerance [84]. *Rht-B1b* and *Rht-D1b*, the most important and common semi-dwarfing genes, encode GA-insensitive forms of DELLA proteins that likely have a reduced affinity for the GA receptor in wheat [85]. It has been reported that the *Rht-B1b* and *Rht-D1b* mutant alleles are not responsive to GA at warmer temperatures but are responsive at colder temperatures (Table 1) [86]. This suggests that *Rht-B1b* and *Rht-D1b* play vital roles in response to cold stress.

The phytohormone jasmonic acid (JA) and its methyl ester, methyl jasmonate (MJ), act as signaling molecules in response to environmental stimuli. Cold stress rapidly increases endogenous JA levels by up-regulating the expression of JA biosynthesis genes, such as *LIPOXYGENASE 1* (*LOX1*), *ALLENE OXIDE SYNTHASE 1* (*AOS1*), *ALLENE OXIDE CYCLASE 1* (*AOC1*), *JASMONATE RESISTANT 1* (*JAR1*) in *Arabidopsis* and *OsLOX2*, *OsAOS*, *OsAOC*, *Oryza sativa 12-OXOPHYTODIENOATE REDUCTASE 1* (*OsOPR1*) in rice (Table 1) [56,87]. The accumulation of JA induced by cold stress is due to the repression of ICE1 by JASMONATE ZIM-DOMAIN 1/4 (JAZ1/4), repressors of jasmonate signaling, resulting in the induction of *CBFs* expression in *Arabidopsis* [87]. Wheat *TaJAZ* genes are up-regulated in response to low temperatures (Table 1) [88]. Additionally, endogenous JA levels increase under cold stress in wheat [89]. Exogenous MJ treatment tends to up-regulate of the transcription of *COR* genes, such as *WCS19* and *WCS120,* and increase the activity of superoxide dismutase (SOD) and peroxidase (PO) to promote wheat cold tolerance [90,91]. Rice HAN1 (“han” means “chilling” in Chinese), which functions as an oxidase to reduce the accumulation of the active to inactive, decreases the expression of *CBF/DREB1s* in rice under cold stress [92]. *Arabidopsis OPR3* is one of the major players in the JA biosynthesis pathway. Transgenic wheat plants with *AtOPR3*-overexpression have increased the accumulation of JA and improved cold tolerance [93].

Brassinosteroids (BRs) play a vital role in plant development and stress tolerance. *COR* gene expression and cold tolerance in *Arabidopsis* are increased by exogenous BR treatment [94]. Exogenous BR treatment promotes growth recovery of maize seedlings following chilling treatment [95] and increases cold tolerance in winter rye and winter wheat [96,97]. BRASSINOSTEROID INSENSITIVE 2 (BIN2) negatively regulates the freezing tolerance in *Arabidopsis* [98]. Knockout mutants of *Oryza sativa GLYCOGEN SYNTHASE KINASE 3-LIKE GENE 1* (*OsGSK1*), an ortholog of *Arabidopsis BIN2*, show enhanced cold tolerance (Table 1) [99]. The expression of *T. aestivum SHAGGY KINASE 5* (*TaSK5*), an abiotic stress-inducible *GSK3*/SHAGGY-like kinase in wheat, is induced at the early stages of cold acclimation (Table 1) [100]. The *BRASSINOSTEROID-INSENSITIVE 1* (*BRI1*) encodes a transmembrane receptor kinase as a BR receptor. Its mutation results in defective BR signaling and increases cold stress tolerance in *Arabidopsis* (Table 1) [101]. The enhanced expression of its wheat homologous *TaBRI1* in *Arabidopsis* leads to better cold tolerance than the wild-type plants by maintaining membrane integrity [102]. Furthermore, overexpression of *TaBRI1* in *Arabidopsis* and the ortholog of *BRI1* in rice or barley increases the silique size and seed yield [103,104]. Therefore, *TaBRI1* is involved in cold tolerance and is a suitable gene for improving crop yields under conditions of extreme environmental stress.

**Table 1:** List of phytohormones in response to cold stress.

Item	Gene	Function of Gene	Regulated by Cold Stress	Reference
Auxin	*OsYUCCA2/3/6/7*	Important gene in Auxin/IPA (indole-3-pyruvic acid) biosynthesis	Up-regulated	[56]
	*OsGH3-1/2/5/6/11*	Auxin/IAA (indole-3-acetic acid) catabolism-related genes	Down-regulated	[56]
	*ARFs*	Regulate the expression of auxin-responsive genes	Up-regulated	[60]
	*TaARFs*	Regulate the expression of auxin-responsive genes	Up-regulated	[61]
ABA	*TaSnRK2.3/2.4/2.8*	Important serine/threonine protein kinase in ABA signaling network	Up-regulated	[66,67,68]
Ethylene	*TaERF1*	A member of the ethylene response factor subfamily of ERF/AP2 transcription factor family	Up-regulated	[78]
	*TaPIE1*	Pathogen-induced ethylene response factor to active stress-related genes	Up-regulated	[79]
Gibberellin	*GRF5*	Growth regulating factor encoding transcription activator.	Up-regulated	[83]
	*SLR1*	A gene that encodes the rice DELLA protein to active *OsGA2ox1* expression	Up-regulated	[84]
	*Rht-B1b*, *Rht-D1b*	The most important and widely used semi-dwarfing genes	Up-regulated	[86]
Jasmonic acid	*LOX1*, *AOS1*, *AOC1*, *JAR1*	JA biosynthesis genes in *Arabidopsis*	Up-regulated	[56]
	*OsLOX2*, *OsAOS*, *OsAOC*, *OsOPR1*	JA biosynthesis genes in rice	Up-regulated	[87]
	*TaJAZs*	The repressors of jasmonate signaling	Up-regulated	[88]
Brassinosteroids	*OsGSK1*	BR negative regulator	Up-regulated	[99]
	*TaSK5*	An abiotic stress-inducible *GSK3* in wheat	Up-regulated	[100]
	*TaBRI1*	BR receptor	Up-regulated	[101]

## 4. ROS and Cold Stress

Abiotic stresses typically increase ROS levels, including hydrogen peroxide (H_2_O_2_), superoxide radical (O_2_^•−^), hydroxyl radical (OH•), and singlet oxygen (^1^O_2_), all of which are toxic to plant cells [105,106,107]. Several pieces of evidence suggest that plant responses to cold stress are directly linked to ROS signaling [108,109,110,111]. It has been proven that low-temperature conditions depress the activities of ROS-scavenging enzymes, such as ascorbate peroxidase (APX), catalase (CAT), superoxide dismutase (SOD), dehydroascorbate reductase (DHAR), monodehydroascorbate reductase (MDHAR), glutathione S-transferase (GST), glutathione reductase (GR), and peroxiredoxin (PRX). These cold-regulated antioxidant enzymes play a key role in enhancing cold tolerance [107,112,113]. The H_2_O_2_ contents of ‘*dongnongdongmai1′* (‘*dn1′*), a winter wheat variety, are significantly increased under cold stress. Additionally, ABA treatment enhances cold tolerance in wheat by increasing the activities of TaSOD, TaAPX, TaCAT, TaGR, TaDHAR, and TaMDHAR [107]. The ABA-stress-ripening (ASR), which functions as a transcription factor, can be induced by low temperatures [114]. The levels of ROS and the activities of antioxidant enzymes under abiotic stress are regulated by ASRs, suggesting that ASR plays an important role in regulating ROS homeostasis [87,115]. Ectopic expression of the cold-induced *OsASR1* gene exhibits enhanced cold tolerance in transgenic rice plants [116]. It has been reported that *TaASR* genes respond strongly to low temperatures [117]. In addition, overexpression of *TaASR1-D* confers enhanced antioxidant capacity and stress tolerance in transgenic wheat plants [118]. *T. aestivum GTP-BINDING PROTEIN β SUBUNIT LIKE GENE* (*TaGPBL*), the first G-protein gene in wheat, contributes to cold stress response. *TaGBPL* overexpression reduces the activity of cold-responsive genes and reduces the activities of ROS scavengers and producers under cold stress [119].

## 5. Cold-Induced Epigenetic Processes and Elements

Epigenetic mechanisms play an important role in response to cold stress. The plant epigenome is highly dynamic, and cold stress can quickly reshape genome-wide epigenetic modifications [120]. Changes in DNA methylation and histone modification and the regulation of epigenetic elements, such as small RNA (sRNA) and long noncoding RNA (lncRNA), are the key modulators of plant stress responses [121,122].

**Figure 2:**
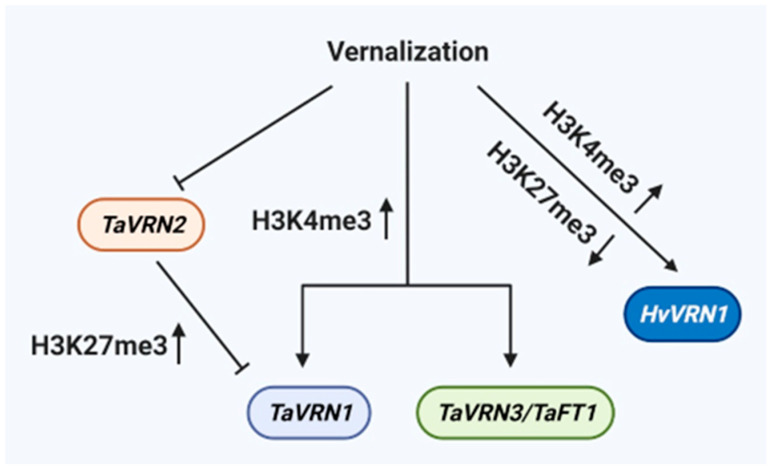
DNA methylation is essential for vernalization pathway in wheat and barley. The expression of *TaVRN2* is down-regulated by vernalization. *TaVRN2* represses the expression of *TaVRN1* by increasing the level of H3K27me3 at *TaVRN1* promoter. Furthermore, vernalization causes an enrichment in the level of H3K4me3 at the *TaVRN1* and *TaVRN3/TaFT1* promoters to up-regulate their expression. In addition, the level of H3K4me3 is up-regulated and the level of H3K27me3 at the *HvVRN1* promoter is down-regulated by vernalization to increase its transcription.

The proteins containing methyl-CpG-binding domain (MBD) can recognize DNA methylation. TaMBD6, including a typical MBD domain at the N-terminal, is induced by prolonged chilling in wheat, indicating that the protein is potentially involved in recognizing DNA methylation during vernalization [123]. Wheat requires various vernalization genes in response to cold stress to adjust floral initiation, such as *T. aestivum VERNALIZATION 1* (*TaVRN1*), *TaVRN2*, *TaVRN3/FLOWERING LOCUS T1* (*TaFT1*), *TaVRN-D4*, and *VERNALIZATION-RELATED 2* (*VER2*) [124,125]. In wheat and barley (*Hordeum vulgare*), the *TaVRN1*, *HvVRN1*, *TaVRN2*, and *TaVRN3/TaFT1* gene are regulated by epigenetic modification (Figure 2). Two histone modification markers include histone 3 lysine 4 trimethylation (H3K4me3), which is a modification associated with active gene transcription, and H3K27me3, which is a modification associated with gene repression. Vernalization enriches H3K4me3 levels at the *TaVRN1* and *TaVRN3/TaFT1* promoters (Figure 2), while no significant changes are observed in H3K27me3 levels at the same regions of the *TaVRN1* and *TaVRN3/TaFT1* promoters in winter wheat. Furthermore, *TaVRN1* and *TaVRN3/TaFT1* are up-regulated by vernalization to accelerate floral transition in winter wheat [126]. *TaVRN2*, a dominant repressor of flowering, is down-regulated by vernalization [127]. Increased levels of H3K27me3 at the *TaVRN1* promoter explain the repression of *TaVRN2* gene expression in winter wheat (Figure 2) [126]. Before cold (vernalization), the increased levels of H3K27me3 at the *HvVRN1* chromatin reduce the transcription of *HvVRN1* in barley. Vernalization increases levels of H3K4me3, the active histone modification marks, and decreases levels of H3K27me3 at *HvVRN1* (Figure 2) [128]. The novel transcript *TaVRN1 ALTERNATIVE SPLICING* (*VAS*), induced by vernalization, functions as a lncRNA derivative from the sense strand of the *TaVRN1* gene to regulate *TaVRN1* transcription during the flowering of winter wheat [129]. Additionally, *TaVRN1* is the earliest target of *TaVRN-D4* among the *TaVRN1*, *TaVRN2*, and *TaVRN3* genes [130]. *VER2* encodes a jacalin-like lectin and promotes *TaVRN1* upregulation by physically interacting with the RNA-binding protein GLYCINE-RICH RNA-BINDING PROTEIN 2 (TaGRP2) after prolonged cold exposure [131]. However, whether the expression of *TaVRN-D4* and *VER2* is associated with DNA methylation requires further study.

Histone acetylation is up-regulated in cold-responsive genes like ZmDREB1 in maize under cold stress [132]. Additionally, cold stress induces higher levels of histone acetylation in the *OsDREB1b* promoter [133]. The level of acetylation is decreased by the up-regulated expression of *HISTONE DEACETYLASEs* (*HDACs*) in maize during cold acclimation [132]. MicroRNA (miRNA) is a class of sRNA that plays a critical role in plant growth and development. miRNA398 (miR398) participates in regulating plant responses to low temperatures in winter turnip rape (*Brassica rapa* L.) [134]. Additionally, the expression of wheat miR398 (tae-miR398) decreases in response to low temperature [135]. It is reported that tae-miR398 regulates cold tolerance by downregulating its target, *COPPER-ZINC SUPEROXIDE DISMUTASE 1* (*CSD1*). Furthermore, the expression of CSD1 is indirectly regulated by lncRNAs (lncR9A, lncR117, and lncR616). The regulation of miR398 induces a regulatory loop that is critical for cold tolerance in wheat [135]. Genome-wide association studies and annotations should be performed to outline the intricately epigenomic landscape, particularly in cereal crops subject to cold stress.

## 6. Conclusions and Perspectives: Improving Cold Tolerance in Wheat

Global food security is a problem of worldwide importance. The rapid increased population and unpredictable climatic events highlight the need to increase crop productivity. Understanding the perception and signaling cascades activated by cold stress response can help develop new technologies that can alleviate yield losses triggered by cold stress. Advances in molecular technologies and a rapidly expanding knowledge of the mechanisms regulating wheat response to cold stress will contribute to improvements in the efficiency of cereal crops.

Phytohormones are dominating regulatory factors of plant growth, development, and signaling networks involved in various abiotic stress responses. This indicates that phytohormones are associated with the cross-talk between environmental stress signals and plant growth. In addition, a growing body of evidence suggests the vital role of the ROS signaling pathway in plant development and stress response in wheat. However, the regulatory mechanisms of plant hormones and ROS in response to cold stress at the biochemical level are still poorly understood. Building comprehensive regulation networks in phytohormones, ROS signaling, and cold tolerance in wheat requires a combination of transcriptomes, proteomics, and metabolomics methods while analyzing mutants and protein–protein interactions.

Systematic research into epigenetic mechanisms in response to abiotic stress, including cold stress, heat stress, drought stress, and salt stress, must be performed under field conditions where multiple stress factors frequently coexist. Inheritable epigenetic processes and elements such as sRNA and lncRNA regulatory mechanisms, histone modification, and DNA methylation could provide within-generation and trans-generational stress memory. More powerful and versatile tools are needed to study epigenetic mechanisms in cereals like wheat in a trans-generational memory context since these epigenetic variations could improve stress tolerance in the offspring.

To successfully develop varieties equipped for cold stress, it is necessary to identify the extent of genetic variation for these traits in wheat. Therefore, future work must identify core components involved in the wheat cold signaling pathway that improve cold tolerance in wheat and increase its production in cold temperatures.
